# The Role of the Grass Snake *Natrix natrix* (Reptilia, Colubridae) in the Transfer of Polyunsaturated Fatty Acids from Aquatic to Terrestrial Ecosystems to Land

**DOI:** 10.1134/S1607672923700564

**Published:** 2023-12-08

**Authors:** Yu. Yu. Dgebuadze, L. A. Neymark, I. V. Bashinskiy, N. N. Sushchik, A. E. Rudchenko, M. I. Gladyshev

**Affiliations:** 1 Severtsov Institute of Ecology and Evolution, Russian Academy of Sciences, Moscow, Russia; 2 Moscow State University, Moscow, Russia; 3 Institute of Biophysics, Federal Research Center “Krasnoyarsk Science Center,” Siberian Branch, Russian Academy of Sciences, Krasnoyarsk, Russia; 4 Siberian Federal University, Krasnoyarsk, Russia

**Keywords:** eicosapentaenoic (EPA) acid, docosahexaenoic (DHA) acids, amphibians, reptiles, organic matter transfer

## Abstract

As a result of analyses of fatty acid (FA) composition in the grass snake *Natrix natrix* and its food objects, tadpoles and metamorphs of two amphibian species: the moor frog *Rana arvalis* and the Pallas spadefoot toad *Pelobates vespertinus*, it was shown for the first time that the high total content of eicosapentaenoic (EPA) and docosahexaenoic (DHA) acids in the biomass of the snakes indicates its important role in the transfer of these essential substances from aquatic ecosystems to land. It was found that, since food sources of DHA in terrestrial ecosystems are absent, its high level in *R. arvalis* metamorps and grass snakes may be provided only by synthesis from biochemical precursors contained in food of aquatic origin.

The composition of polyunsaturated fatty acids (PUFAs) in vertebrates almost entirely depends on the food consumed from water bodies, since these substances are synthesized mainly by aquatic microorganisms. For this reason, PUFAs are often used as biomarkers in assessing the transfer of biomass from aquatic to terrestrial ecosystems [1]. Basically, this transfer occurs when aquatic organisms are eaten by terrestrial ones, or when the habitat of amphibiotic animals with aquatic larvae—amphibians and insects—changes. Currently, the role of reptiles in the transfer of substances remains poorly understood. In Russia, the most promising reptile species for research in this area is the common grass snake (*Natrix natrix* (Linneaus, 1758)). This snake feeds predominantly on aquatic organisms, being itself a component of the diet of many terrestrial animals, and may contribute to the transfer of the biomass from aquatic to terrestrial ecosystems.

For example, in the Volga region, grass snake was found in the diet of 54 species of vertebrates, mainly birds (for example, Ciconiiformes, Falconiformes), and mammals (for example, insectivores and carnivores) [2]. The diet of the grass snake itself is dominated by amphibians. For instance, in the Volga basin, according to different studies, they account for 70.8 [3] to 92.8% [4] of the diet. Cases of eating of mammals, reptiles, and birds by grass snakes were reported in many studies (for example, [5, 6]); however, in total they account for no more than 2% of the diet by biomass [7]. Adult snakes more often eat metamorphosed frogs than tadpoles. The maximum proportion of tadpoles in the grass snake’s diet (33.3%) was reported by Klenina [3]. Usually, the diet of grass snakes is dominated by the marsh frog *Pelophylax ridibundus* (Pallas, 1771), the moor frog *Rana arvalis* Nilsson, 1842, and the common frog *Rana temporaria* Linneaus, 1758. In the Penza Region, the grass snake’s diet was dominated by the moor frog (61%) and the marsh frog (20%) [8]. It can be assumed that the grass snake does not show significant selectivity when choosing between different frog species and eats those that are most often found near the water edge.

The purpose of this work was to assess the role of the grass snake in the transfer of polyunsaturated fatty acids from the aquatic environment to land. The study was performed in the Penza Region, in the vicinity of the Privolzhskaya Lesostep State Nature Reserve, on small floodplain water bodies of the Khopyor River (52°48′58.4″ N, 44°27′40.4″ E) in 2015 and 2018–2019.

To analyze the PUFA content, muscle tissue samples from seven snakes were collected. The samples were minced and fixed in 2–3 mL of a chloroform : methanol mixture (2 : 1). Similar samples were taken in 2015 from the food items of grass snakes—tadpoles and metamorps of two common amphibian species, the moor frog *Rana arvalis* (10 specimens of tadpoles and 15 specimens of metamorps) and the Pallas spadefoot toad *Pelobates vespertinus* (8 specimens of tadpoles and 15 specimens of metamorps). Biochemical analysis was performed according to the standard methods using gas-liquid chromatography combined with mass spectrometry [9].

Multivariate correspondence analysis of the FA composition of the studied animals revealed significant differences in the first (largest) dimension between tadpoles of both species and *P. vespertinus* metamorps, on the one hand, and *R. arvalis* metamorps and grass snakes, on the other hand (Fig. 1). The differences between the samples of these species were largely due to differences in the levels of 18:4*n*-3 FA and ∑16PUFA (the sum of 16-atom PUFAs) (markers of microalgae [10]), on the one hand, and 24:1 (markers of higher plants [10]) and C18-20 monounsaturated FAs (de novo synthesis products in animals [10]), on the other hand (Fig. 1). In addition, in the second dimension, significant differences were found between *R. arvalis* tadpoles, on the one hand, and *P. vespertinus* tadpoles and metamorps, on the other hand, which were mostly due to the differences in the levels of 12:0 and 18:4*n*-3 FAs in their biomass (Fig. 1). The 18:4*n*-3 FA, which was present in relatively high amounts in *P. vespertinus* tadpoles and metamorphs and was absent in grass snakes (Table 1), is a marker of cryptophyte and chrysophyte algae [10].

**Fig. 1.**
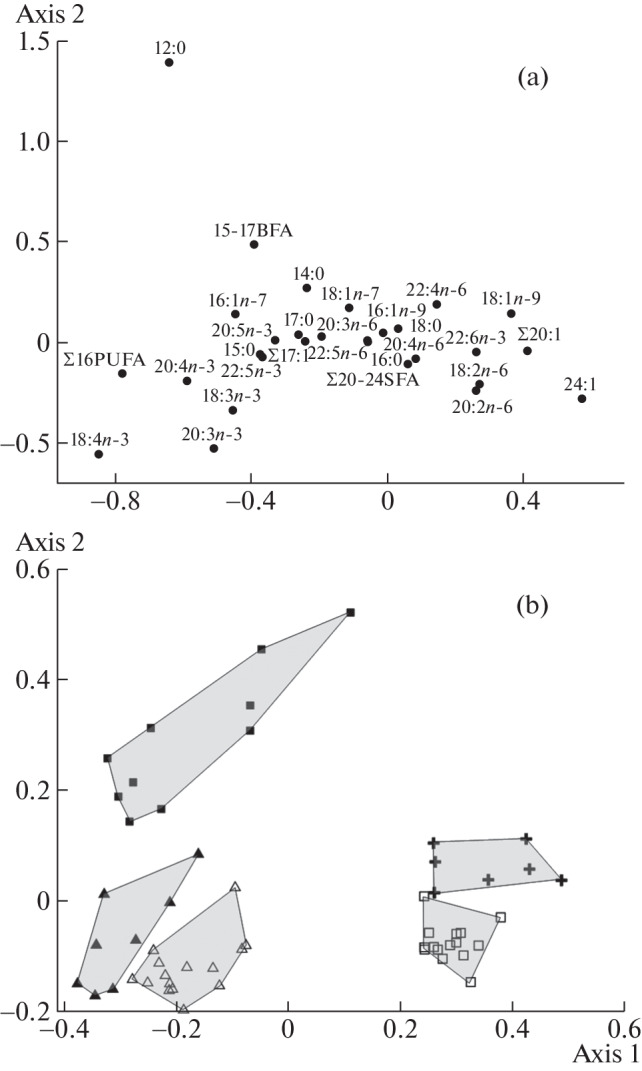
Canonical multivariate correspondence analysis of fatty acid fractions (a) in amphibians and snakes biomass (b): shaded and empty squares—tadpoles and metamorphs of *R. arvalis*, respectively; shaded and empty triangles—tadpoles and metamorphs of *P. vespertinus*, respectively; crosses—snakes *N. natrix*. Oxbows of the Khoper River, Privolzhskaya Lesostep State Nature Reserve (Penza Region), June 2015 (amphibians) and 2018–2019 (grass snakes). The proportion of explained variance (inertia) along Axes 1 and 2 is 45.6 and 16.9%, respectively; χ^2^ = 862, number of degrees of freedom 1512.

**Table 1.** Mean content of quantitatively significant fatty acids (% of total fatty acids ± standard error) and their sum (mg/g wet weight) in the biomass of tadpoles and metamorphs of *P. vespertinus* and *R. arvalis* and grass snake *N. natrix*. The mean values with normal distribution marked with the same letter (by rows) have no significant differences (P > 0.05) according to Tukey’s HSD test; the mean values marked with * (non-normal distribution) were compared by non-parametric Kruskal–Wallis test. Oxbows of the Khoper River, Privolzhskaya Lesostep State Nature Reserve (Penza Region), June 2015 (amphibians) and 2018–2019 (grass snakes)

F.A.	*R. arvalis* tadpoles	*P. vespertinus* tadpoles	*R. arvalis* metamorphs	*P. vespertinus* metamorphs	*N. natrix*
12:0*	1.1 ± 0.13^A^	0.1 ± 0.00^AB^	0.1 ± 0.01^BC^	0.1 ± 0.00^V^	0.0 ± 0.00^C^
14:0*	1.7 ± 0.26^A^	0.8 ± 0.06^AB^	0.8 ± 0.07^BC^	1.3 ± 0.18^AC^	0.3 ± 0.03^V^
15:0	0.8 ± 0.03^A^	0.8 ± 0.06^A^	0.4 ± 0.03^V^	1.1 ± 0.05^C^	0.3 ± 0.03^V^
16:0	22.2 ± 0.60^A^	22.1 ± 0.52^A^	20.6 ± 0.41^AB^	19.9 ± 0.44^V^	15.9 ± 0.53^C^
16:1*n*-9	0.7 ± 0.11^AB^	0.7 ± 0.06^AB^	0.8 ± 0.03^A^	0.5 ± 0.05^BC^	0.3 ± 0.07^C^
16:1*n*-7	5.8 ± 0.36^A^	5.3 ± 0.33^AC^	1.8 ± 0.21^V^	4.7 ± 0.22^C^	1.8 ± 0.26^V^
15-17BFA	2.5 ± 0.08^A^	1.0 ± 0.07^BD^	0.4 ± 0.03^C^	1.2 ± 0.08^V^	0.8 ± 0.11^D^
Σ16PUFA	0.2 ± 0.04^AC^	0.2 ± 0.04^A^	0.01 ± 0.01^V^	0.3 ± 0.04^A^	0.04 ± 0.02^BC^
17:0	1.4 ± 0.03^A^	1.2 ± 0.03^V^	0.7 ± 0.03^C^	1.5 ± 0.04^A^	0.7 ± 0.06^C^
Σ17:1	0.6 ± 0.06^AB^	0.4 ± 0.03^BC^	0.3 ± 0.02^C^	0.7 ± 0.04^A^	0.4 ± 0.04^C^
18:0	10.4 ± 0.28^A^	9.6 ± 0.53^AB^	9.4 ± 0.40^AB^	8.1 ± 0.46^V^	10.1 ± 0.24^A^
18:1*n*-9	12.7 ± 2.34^AB^	8.4 ± 0.55^V^	16.8 ± 0.92^A^	9.4 ± 0.62^V^	22.5 ± 1.73^C^
18:1*n*-7	6.8 ± 0.33^A^	5.5 ± 0.25^V^	3.4 ± 0.08^C^	5.3 ± 0.19^V^	6.3 ± 0.51^AB^
18:2*n*-6	5.1 ± 0.30^A^	6.8 ± 0.32^A^	14.4 ± 0.55^V^	10.2 ± 0.20^C^	13.0 ± 0.65^V^
18:3*n*-3	2.7 ± 0.27^A^	7.1 ± 0.92^V^	2.4 ± 0.36^A^	6.9 ± 0.50^V^	1.9 ± 0.12^A^
18:4*n*-3*	0.04 ± 0.03^AB^	0.2 ± 0.03^AC^	0.01 ± 0.01^V^	0.3 ± 0.09^C^	0.0 ± 0.00^V^
Σ20-24SFA	1.0 ± 0.12^AB^	1.1 ± 0.10^AB^	1.4 ± 0.14^A^	1.0 ± 0.11^AB^	0.7 ± 0.07^V^
Σ20:1*	0.3 ± 0.07^AB^	0.3 ± 0.04^V^	0.5 ± 0.03^AC^	0.4 ± 0.03^AB^	1.2 ± 0.05^C^
20:2*n*-6	0.1 ± 0.04^A^	0.2 ± 0.01^AC^	0.4 ± 0.02^V^	0.3 ± 0.01^C^	0.4 ± 0.06^V^
20:3*n*-6	0.9 ± 0.07^A^	0.8 ± 0.02^AB^	0.9 ± 0.04^A^	0.8 ± 0.02^AB^	0.6 ± 0.02^V^
20:4*n*-6	6.5 ± 0.31^A^	6.2 ± 0.31^A^	8.7 ± 0.38^V^	8.5 ± 0.46^V^	9.0 ± 0.60^V^
20:3*n*-3*	0.1 ± 0.03^A^	0.7 ± 0.08^BC^	0.2 ± 0.01^AB^	0.8 ± 0.04^C^	0.1 ± 0.01^A^
20:4*n*-3	0.5 ± 0.08^AB^	0.7 ± 0.05A	0.2 ± 0.01^V^	0.7 ± 0.16^A^	0.1 ± 0.02^V^
20:5*n*-3	6.3 ± 0.40^A^	8.8 ± 0.22^V^	3.8 ± 0.15^C^	5.3 ± 0.15^D^	2.7 ± 0.30^E^
22:4*n*-6	0.4 ± 0.03^A^	0.3 ± 0.05^V^	0.3 ± 0.03^AB^	0.3 ± 0.02^V^	0.6 ± 0.05^C^
22:5*n*-6*	0.5 ± 0.04^A^	0.1 ± 0.01^V^	0.2 ± 0.02^V^	0.7 ± 0.21^A^	0.6 ± 0.08^A^
22:5*n*-3*	3.9 ± 0.65^ABC^	5.6 ± 0.19^AC^	2.1 ± 0.08^V^	4.3 ± 0.21^AC^	2.2 ± 0.20^BC^
22:6*n*-3	3.6 ± 0.36^A^	3.1 ± 0.10^A^	6.2 ± 0.30^V^	3.3 ± 0.20^A^	5.9 ± 0.55^V^
24:1*	0.3 ± 0.05^A^	0.5 ± 0.07^A^	1.8 ± 0.26^V^	0.5 ± 0.06^A^	0.7 ± 0.10^AB^
Total FA	2.8 ± 0.38^A^	1.6 ± 0.05^A^	6.1 ± 0.39^V^	5.6 ± 0.39^V^	6.6 ± 0.96^V^

Comparison of the statistical significance of differences in the levels of individual acids (Table 1) made it possible to clarify the results of multivariate analysis. Tadpoles of both amphibian species and *P. vespertinus* metamorps had significantly higher levels of bacterial acids 15:0, 15-17BFA (branched acids), 17:0 and saturated acid 16:0, which is synthesized by animals themselves, as well as 16:1*n*-7 and 20:5*n*-3 (EPA) FAs, which are synthesized by diatoms.

In turn, *R. arvalis* metamorps and grass snakes had significantly higher levels of essential linoleic acid 18:2*n*-6, a marker of food of terrestrial origin, than tadpoles of both species and *P. vespertinus* metamorps (Table 1). In addition, *R. arvalis* metamorps and grass snakes had significantly higher levels of 20:2*n*-6, which is synthesized from the essential FA 18:2*n*-6, than tadpoles of both species and *P. vespertinus* metamorps (Table 1). In *R. arvalis* metamorps and grass snakes, the level of 22:6*n*-3 (DHA) was significantly higher than that in tadpoles of both species and *P. vespertinus* metamorps (Table 1).

When comparing the FA composition of tadpoles of each species with metamorps, it should be noted that the level of linoleic acid and arachidonic acid 20:4*n*-6 (markers of food of terrestrial origin [10]) in tadpoles was significantly lower than in metamorps (Table 1). Conversely, the levels of EPA, a marker of food of aquatic origin [10], in tadpoles were higher than in metamorps (Table 1).

Despite the fact that the grass snake samples were taken in other years than the amphibian samples, when comparing the compositions of their FAs, one can note a significantly lower level of 16:0, 15-17BFA, and EPA and a significantly higher level of 18:1*n*-9 and 22 :4*n*-6 in grass snakes (Table 1).

Measurements of the content of physiologically significant PUFAs, EPA, and DHA, in biomass (mg/g wet weight), characterizing the quality of organic matter transmitted on food webs, gave the following results. The maximum EPA content was found in *P. vespertinus* metamorps (Fig. 2). The maximum DHA content was detected in *R. arvalis* metamorps and grass snakes (Fig. 2). The total content of EPA + DHA in metamorps of both species and grass snakes was significantly higher than in tadpoles (Fig. 2), which indicates the accumulation of these essential PUFAs in the biomass of amphibians during metamorphosis, as well as in the biomass of grass snakes as top-level consumers in relation to amphibians.

**Fig. 2.**
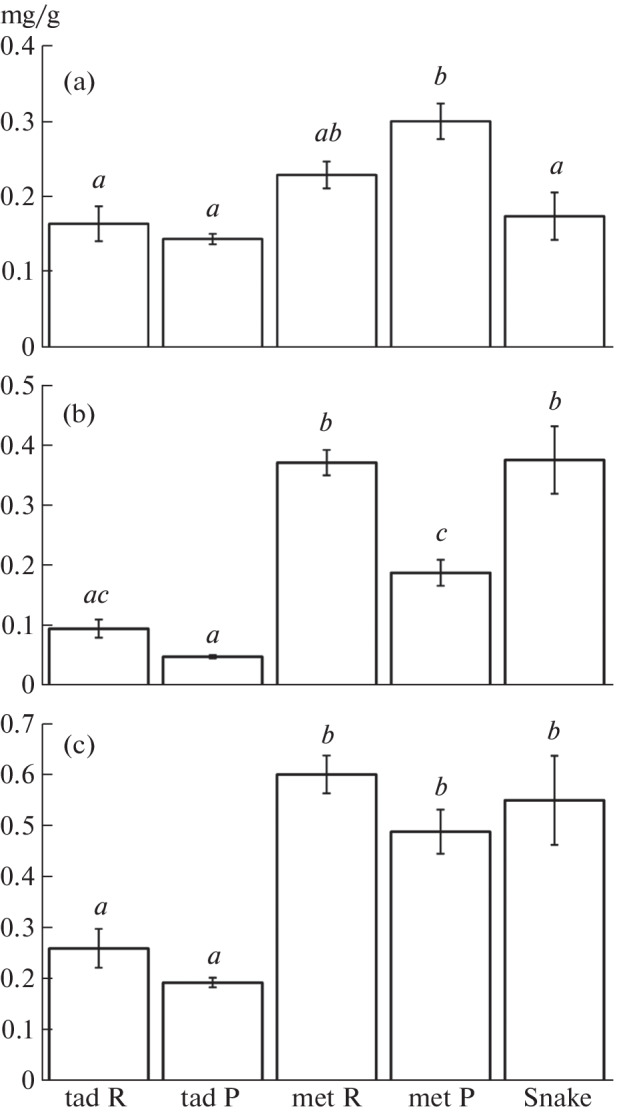
Mean content (mg/g wet weight ± standard error) in the biomass of amphibians and grass snakes: (a) eicosapentaenoic acid, (b) docosahexaenoic acid, and (c) their sum. Designations: tad R, *R. arvalis* tadpoles; tad P, *P. vespertinus* tadpoles; met R, *R. arvalis* metamorps; met P, *P. vespertinus* metamorphs; snakes, *N. natrix*. Oxbows of the Khoper River, Privolzhskaya Lesostep State Nature Reserve (Penza Region), June 2015 (amphibians) and 2018–2019 (grass snakes).

Thus, the use of fatty acids as biomarkers (higher levels of the essential linoleic acid 18:2*n*-6) showed that *R. arvalis* metamorps have a higher proportion of terrestrial food in their diet than *P. vespertinus* metamorps. Differences in markers may be associated with the development of these two species, which differs in timing and duration [11]. This is most likely due to the fact that *R. arvalis* tadpoles complete metamorphosis earlier and begin to emerge from the water body in early June, whereas the emergence of *P. vespertinus* begins at the end of June and continues until August. Accordingly, *R. arvalis* metamorps switch to feeding on terrestrial food earlier than *P. vespertinus* metamorps.

The significantly higher level of DHA in muscle tissue probably indicates that *R. arvalis* metamorps and grass snakes are more active: they move more intensely (faster and/or longer) than *P. vespertinus* metamorps, which is consistent with observations of the lifestyle of these species. *P. vespertinus* are active predominantly at night, whereas *R. arvalis* is also active during the day [12]. Moreover, the levels of the biochemical precursor, EPA, in *R. arvalis* metamorps and grass snakes are lower than those in tadpoles of both amphibian species and *P. vespertinus* metamorps. It can be concluded that, since there are no food sources of DHA in terrestrial ecosystems, the high level of DHA in *R. arvalis* metamorps and grass snakes can only be achieved through synthesis from biochemical precursors probably contained in food of aquatic origin.

The relatively high total content of EPA + DHA in the biomass of grass snakes indicates the important role of this amphibiotic animal in the transfer of these essential PUFAs from aquatic ecosystems to land.

The accumulation of EPA and DHA in the upper components of aquatic and aquatic-terrestrial trophic webs was also found in other ecosystems [13]. In general, the average content of EPA and DHA in the biomass of frogs and grass snakes from 0.2 to 0.8 mg/g wet weight was comparable to the average content of these acids in some fish species of the order Cypriniformes [14].
